# Complete genome sequence of *Brachybacterium* sp. GU-2 (Actinomycetota), isolated from the massive coral *Porites lobata*

**DOI:** 10.1128/MRA.00855-23

**Published:** 2023-11-29

**Authors:** Gaurav G. Shimpi, Pablo De la Vega, Bastian Bentlage

**Affiliations:** 1 Marine Laboratory, University of Guam, Mangilao, Guam, USA; University of Southern California, Los Angeles, California, USA

**Keywords:** coral microbiome, actinomycetes

## Abstract

*Brachybacterium* sp. GU-2 was isolated from the hard coral *Porites lobata* found in Apra Harbor, Guam, Micronesia. This genome sequence will be beneficial to understand the role of actinomycetes in coral holobionts. The *Brachybacterium* genome contains several gene clusters for bioactive compounds, including antibiotics.

## ANNOUNCEMENT

Marine Actinomycetota garnered attention to meet growing pharmaceutical demands for antimicrobial and other bioactive compounds of commercial value (1, 2). The genus *Brachybacterium* (Micrococcales: Dermabacteraceae) comprises ~28 species spanning various ecological niches and have been found associated with scleractinian corals, exhibiting antimicrobial properties (3, 4). Moreover, the species *B. conglomeratum* was part of a beneficial microorganisms of corals consortium used to mitigate heat stress and prevent coral bleaching and mortality (5). Despite the association with corals and putative beneficial roles in coral health, genome sequences and isolates of coral-associated *Brachybacterium* spp. are lacking. We provide a genome sequence from the genus *Brachybacterium* isolated from a coral.

*Brachybacterium* sp. GU-2 was isolated from *Porites lobata* from Finger Reef located in Apra Harbor, Guam (13°44′76″N 144°62′88″E). A ~5-cm coral fragment was sampled into sterile seawater. A portion of the fragment was rinsed and crushed in sterile seawater using mortar and pestle to obtain ~25 mL of homogenized slurry composed of coral tissue and skeleton. Next, 10-fold and 100-fold tissue homogenate dilutions were plated onto marine agar (Marine Broth 2216 plus 1.5% agar; Millipore Sigma, USA) and incubated at 23°C for 4 days. A tiny round, convex yellowish-white colony was picked and inoculated in 5 mL of marine broth and incubated for 48 h at 23°C. Three rounds of sub-culturing were performed, and a single colony was selected for the inoculation of 5 mL marine broth that was allowed to grow for 48 h before DNA extraction.

High-molecular weight (HMW) DNA was extracted using the Monarch HMW DNA Extraction Kit T3060 (NEB, USA). Sequencing libraries were prepared with non-size-selected DNA using a Native Barcoding Kit (SQK-NBD114.96) and a MinION Sequencer (ONT, UK). Bases were called using Guppy v6.3.7 in MinKNOW software (Mk1B 23.04.6; ONT, UK) which also trimmed barcodes. Flye v2.9.2 was used for genome assembly and genome polishing (6). Default parameters were used for all software unless otherwise specified. A total of 298,488 reads were assembled into a circular chromosome of ~3.68 Mb (Table 1). While the standard practice for ONT-based bacterial genome assemblies has been incorporation of Illumina data for error correction, the ﬂow cell and sequencing chemistry (version R10.4.1) employed by us has been suggested to provide error rates of reads < 1% to allow for high-quality assemblies (7, 8). The final assembly was checked for completeness (98.83%) and contamination (0.58%) using CheckM v1.2.2 (9) with the lineage-specific (Actinomycetales) option that used 580 genomes and 286 markers. The *Brachybacterium* genome had a very high GC content (72%), a characteristic of the majority of Actinomycetota (10).

**TABLE 1:** *Brachybacterium* sp. GU-2 genome assembly statistics

NCBI Accession No.	Genome size (bp)	Coverage (×)	ONT Read No.	Read N_50_ (bp)	GC content (%)	No. of^a^				Genome Completeness^b^ (%)
						CDSs	23S	16S	5S	
CP133037	3,684,078	696**×**	298,488	31,270	72	3,312	3	3	3	98.83

^a^ The number of coding sequences (CDSs) and rRNAs was predicted using RAST (11).

^b^ Genome completeness was assessed using a set of 580 *Actinomycetales* genomes and 286 markers by CheckM.

**Fig 1:**
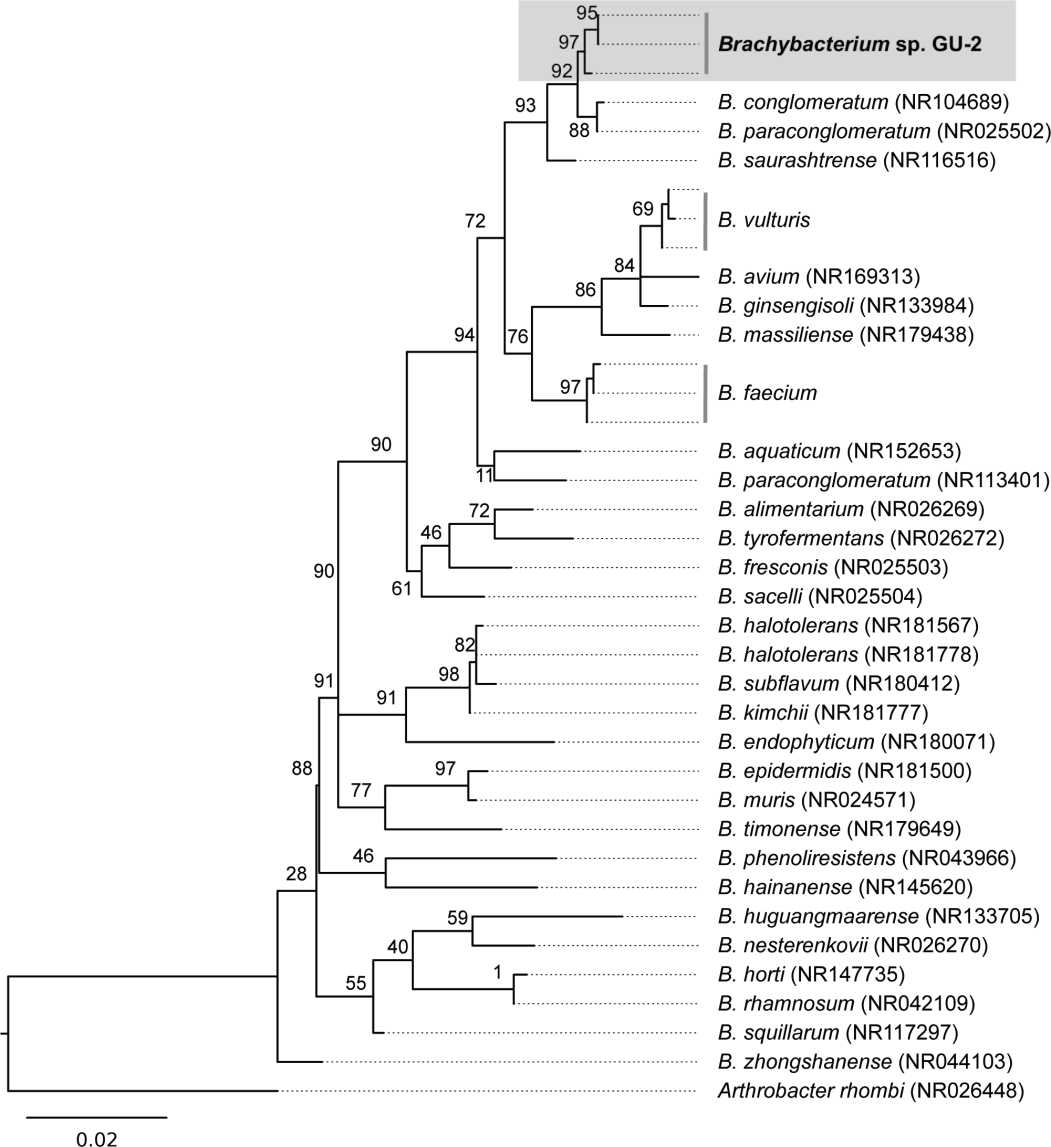
*Brachybacterium* spp. 16S rRNA gene ML phylogeny inferred under the TIM3+I+G model. Bootstrap supports shown on nodes were calculated using 1,000 nonparametric replicates. Scale bar denotes substitutions per site.

The genome was annotated using NCBI’s Prokaryotic Genome Annotation Pipeline (12). A total of 9 rRNAs and 51 tRNAs were identified with three 16S rRNA gene copies. All three 16S rRNA gene copies were combined with publicly available sequencing data and aligned using ClstalW (13) prior to maximum-likelihood (ML) phylogenetic analysis using raxmlGUI 2.0 (14, 15) under the TIM3+I + G model identified by ModelTest-NG (16); *Brachybacterium* sp. GU-2 forms a well-supported monophyletic clade with *Brachybacterium conglomeratum*, *Brachybacterium paraconglomeratum*, and *Brachybacterium saurashtrense* (Fig. 1). The genome sequence provided here represents a resource for understanding the role of Actinomycetota in coral health and has the potential to aid natural product discovery given the presence of several biosynthetic clusters, including, but not limited to, carotenoid and ectoine synthesis, siderophores, and 17 polyketides as well as putative antimicrobial compounds identified by antiSMASH v7.0.1 (17).

## Data Availability

ONT reads were deposited in the NCBI Sequence Read Archive (SRA) under accession number SRR25645445. Genome assembly (CP133037) available through NCBI BioProject number PRJNA1005016.
